# Rapid detection of *Mycobacterium tuberculosis* complex by real-time polymerase chain reaction (PCR) in pulmonary and extra-pulmonary samples in Casablanca, Morocco

**DOI:** 10.11604/pamj.2020.36.134.16652

**Published:** 2020-06-26

**Authors:** Maaloum Fakhreddine, Katfy Khalid, Diraa Othman, Diawara Idrissa, Belabbes Houria, Zerouali Khalid

**Affiliations:** 1 Laboratoire de Microbiologie, Faculté de Médecine et Pharmacie, Université Hassan II de Casablanca, Casablanca, Maroc,; 2 Laboratoire de Bactériologie, Virologie et Hygiène Hospitalière du CHU Ibn Rochd de Casablanca, Casablanca, Maroc,; 3 Faculté des Sciences et Techniques de Santé, Université Mohammed VI des Sciences de la Santé, Casablanca, Maroc

**Keywords:** *Mycobacterium tuberculosis* complex, IS6110, real-time polymerase chain reaction, tuberculosis

## Abstract

**Introduction:**

the laboratory diagnosis of tuberculosis (TB) relies mainly on conventional techniques. However, it either lacks sensitivity or it is time-consuming. This study aims to evaluate the use of real-time polymerase chain reaction (PCR) targeting IS6110 for Mycobacterium tuberculosis (TB) Complex (MTBC) in the routine diagnosis of TB in our laboratory.

**Methods:**

clinical samples were collected from the laboratory of bacteriology at Ibn Rochd University Hospital in Casablanca Morocco. Real-time polymerase chain reaction (PCR) results were compared to AFB smear and culture on Löwenstein-Jensen (LJ) solid media. Sensitivity, specificity, positive and negative predictive value (PPV and NPV) with 95% confidence intervals were calculated using GraphPad Prism.

**Results:**

on 171 clinical samples, the study showed positivity of microscopy, culture and real-time PCR for M. TB complex as 19%, 31%, and 32% respectively. Sensitivity, specificity, PPV and NPV for real-time PCR in pulmonary samples were 95.2%, 95.4%, 90.91% and 97.65% respectively. For extra-pulmonary samples, they were: 72.7%, 90.32%, 72.7%, and 90.3%.

**Conclusion:**

our study shows the effectiveness of using real-time PCR IS6110 in pulmonary and extra pulmonary samples. Future multicentric studies could seek to evaluate the place of this technique on routine diagnosis for better management of TB in Morocco.

## Introduction

Tuberculosis (TB) is one of the most frequent causes of death worldwide and is the leading infectious killer globally, surpassing HIV and malaria. It caused 1.7 million deaths in 2016 (including 400 000 people with HIV). More than 95% of TB deaths occur in low-and-middle-income countries. Seven countries account for 64% of the overall number, with India leading the count, followed by Indonesia, China, Philippines, Pakistan, Nigeria, and South Africa. The WHO also estimates that 1 million children have developed TB among who 170.000 have died (excluding those with HIV) [1]. In Morocco, 30.636 cases of TB, of all forms (28,955 new cases and 1,681 cases of relapses) were diagnosed in 2015, with an incidence rate of 89 cases per 100.000 inhabitants. Between 2000 and 2015, the overall rate decreased by 17%, while the smear-positive pulmonary form decreased by 20%. 44% of TB cases confirmed bacteriologically, compared to 56% of these, which were diagnosed in line with clinical criteria [2].

Laboratory diagnosis of TB relies mainly on acid-fast bacilli (AFB) smear for developing countries. This technique is quick and easy to set up, and it has a low and variable sensitivity (50-80%) as well as a suboptimal specificity [3, 4]. However, the result of culture followed by phenotypic and biochemical identification remains the gold standard. The disadvantage of this method is usually the time of growth, which can range from 6 to 8 weeks. As a result, recent innovations have been focusing on the development of new molecular biology tools that have the dual advantages of speeding up the diagnosis of TB as well as the detection of anti-TB drug resistance. PCR has been shown to be effective for the diagnosis of TB, mainly because of its short response time and significant sensitivity [5]. PCR has evolved considerably with the introduction of fluorescent probes to become a Real-time PCR. This technique has become increasingly attractive to laboratories wishing to have the diagnosis of active TB in their panel due to its sensitivity gain and dual-specificity for the detection of the causative agent [6]. In fact, for TB diagnosis, multiple commercial and in-house nucleic acid amplification techniques have been developed [7]. For instance, the Xpert RIF/TB, (GeneXpert, Cepheid, Canada) has revolutionized the detection of pulmonary TB [8]. Approved by the WHO, this system is fast and easy to use, but it remains expensive and cannot be ideal in low-income diagnostic structures. However, both the lack of sensitivity in extra-pulmonary specimens and smear negative leave many questions unanswered still [9]. Our study was conducted at Ibn Rochd University Hospital Centre in Casablanca, Morocco, with the objective of applying an in-house real-time PCR to detect MTBC in pulmonary and extra-pulmonary samples.

## Methods

**Sampling and study design:** samples had been collected from mycobacteria laboratory at Ibn Rochd University Hospital in Casablanca-Morocco, for 6 months between July and December 2018. Inclusion criteria for this study consist of collecting all pulmonary and extra-pulmonary specimens from children (0-15 years) and adults patients for diagnosis of TB, patients coming from high-risk units of TB (Pneumology, Phthisiology, Infectious diseases, etc.) with a strong suspicion of TB (Unexplained weight loss, loss of appetite, night sweats, fever) [10], it should be noted that HIV status was unknown. Also, duplicates and non-compliant samples were excluded from the study. All the collected samples were subjected to microscopic examination, culture on LJ solid media and real-time PCR for the detection of MTBC.

**AFB smear:** the presence and quantitation of resistant acid fast bacilli in samples were performed by the heat carbol fuchsin method.

**Sample processing:** samples were digested and decontaminated using the trisodium phosphate technique [11], a procedure used routinely in our laboratory with some modifications [12]. This technique was compared to the modified Petroff’s method to evaluate the impact of decontamination in real-time PCR performance. All samples were cultured in two LJ tubes in parallel. Positive cultures were subjected to biochemical identification using niacin, nitrate, and the heat-resistant catalase test (HRCT).

**DNA extraction:** the boiling method was used to inactivate 300µl of the digested samples [13]. Extraction was carried out by QIAmp DNA Minikit (Qiagen, Hilden, Germany) according to the manufacturer's recommendations, and 150µL of DNA was stored in a sterile tube at -20°C until the use by Real-time PCR.

**Real-time PCR:** a duplex real-time PCR was adopted to target a 123bp fragment highly conserved and repeated region of the IS6110 in MTBC, and a 105bp fragment belonging to a human ERV3 gene used to control the inhibition of the PCR reaction. Amplification was performed with the primer pairs and the probes previously published [14] with some modifications. The final volume of the reaction mixture was 25µL, which contained 1X of TaqMan Universal PCR Master Mix and 5µL of DNA. All Real-time PCR were performed in the CFX 96® real-time thermal cycler (Biorad® Life Science Group, California USA). The PCR amplification protocol was changed as follows: Initial denaturation at 95°C for 10 min followed by 50 cycles of amplification of 95°C for 10 seconds and one minute at 60°C. The validation of PCR results for MTB by IS6110 was considered positive if the Ct value was < 42 and the ERV3 PCR was considered positive if the Ct was < 46.

**Statistical analysis:** sensitivity, specificity, positive, and negative predictive value with 95% confidence intervals were calculated considering culture on solid media as the gold standard method. The proportion of positive results for each test was compared using McNemar’s test. Differences between groups were compared using Chi-square test for proportions and Mann-Whitney U test for continuous variables, with p-values < 0.05 considered statistically significant. Agreement between culture and real time PCR was presented by kappa coefficient. Statistical analyses were performed using GraphPad Prism (version 6.0; GraphPad Software, USA, and the VassarStats online statistical package.

**Ethics statement:** all specimens were collected and received for routine diagnostic purposes and were no longer needed. No private patient information was shared with anyone external to this study. Since patient consent was not required, we did not need Institutional Review Board approval.

## Results

**Figure 1:**
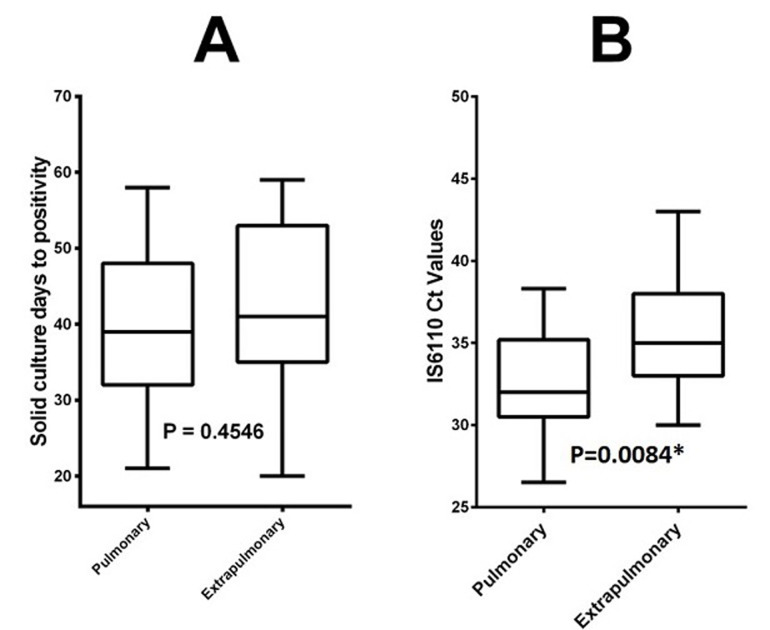
comparison of culture days-to-positivity (A) and IS6110 CT values (B) pulmonary specimens versus extra-pulmonary specimens

Over the study period, the laboratory received 629 samples, of which 171 met our inclusion criteria. Among these 171 samples, 75% were pulmonary origin, AFB smears were positive in 32 cases (18.7%), culture on LJ showed 53 samples as positive (31%) and IS6110 real-time PCR detected 55 MTBC (32%) (Table 1). The comparison of the modified Petroff 's method and trisodium phosphate showed no discordance during the real-time PCR performance. The difference was not statistically significant using the chi-squared test (p > 0.05). AFB Smear results showed an agreement of 32 positive samples with real-time PCR. Another 23 samples showed negative results by smear, but positive findings in real-time PCR. For 48 samples, culture on LJ media showed the same results as real-time PCR. Discrepancies were retained for 5 positive samples in culture that were negative on real-time PCR (Figure 1).

**Table 1:** percentage of positive results from conventional tests and PCR (%) according to the samples

Expectoration	(n=95)	Bronchium Aspiration (n=25)	Tubing gastric (n=9)	BAL* (n=4)	Pus (n=20)	Biologic liquids (n=15)	Biopsy (n=3)	Total (n=171)
AFB	25(26%)	2(8%)	1(11%)	0(0)	2(10%)	1(6%)	1(33%)	32(19%)
Culture	32(33%)	7(28%)	3(33%)	1(25%)	5(25%)	4(26%)	1(33%)	53(31%)
Real-time PCR	34(35%)	8(32%)	3(33%)	0(0)	7(35%)	2(13%)	1(33%)	55(32%)

* Bronchoalveolar lavage

**Figure 2:**
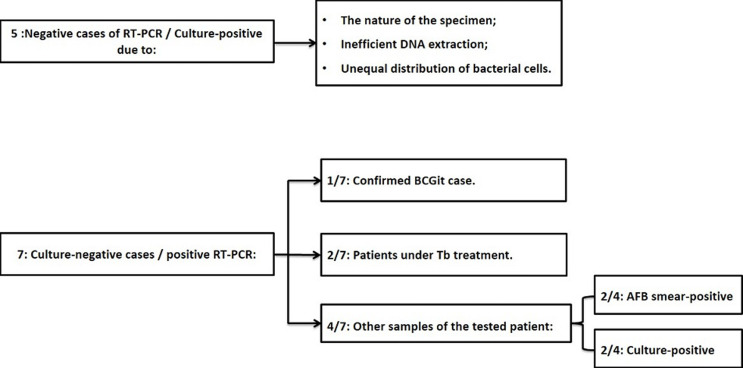
a schema representing discrepancies between culture and real-time polymerase chain reaction (PCR)

Another discordance was detected for 7 positive samples in real-time PCR, which were negative in culture, 3 of these were positive on AFB smear. The remaining 4 were compared to clinical data to decide on their positivity (Figure 2). The correlation between real-time PCR results and culture showed a high degree of agreement κ = 0.840. The sensitivity of comparing real-time PCR in pulmonary specimens to extra-pulmonary specimens was 95.2% (40/42) versus 72.7% (8/11); p < 0.05, the specificity was 95.4% (83/87) versus 90.3% (28/31); p < 0.05, positive predictive value (PPV) with 90.91% vs. 72.73% and negative predictive value ((NPV) with 97.65 % vs. 90.32); p < 0.05. Also, the global sensitivity, specificity, PPV and NPV of our real-time PCR are 90.6%, 94.1%, 87.3%, and 95%. No difference was retained between means of days to positivity for solid culture in pulmonary and extra pulmonary samples [39 (32-48) vs. 41 (35-53); p: 0.45]. Ct values for real-time PCR were statistically significant between pulmonary and extra pulmonary specimens [32 (31-35) vs. 35 (33-38); p: 0.0084)] (Figure 1).

## Discussion

TB is endemic and a major public health problem in Morocco. The global incidence of TB is very high, with nearly 30,000 new cases per year. TB affects young adults and, thus, has a high impact on the socioeconomic status of the country. In fact, 65.9% of total TB cases are between 15 and 45 years of age, with a significant male predilection (59.3%) [15]. Casablanca, the largest Moroccan city, figures almost one-fifth of the total cases recorded in the country [16]. The early and rapid diagnosis of MTB is very important to control and initiate drug treatment regimen. The laboratory diagnosis of TB is based mainly on the microscopic examination by the AFB Smear and MTB culture, which are widely used in of public health center laboratories for routine analyses [17]. In recent years, bacteriological diagnosis of TB has indeed benefited from many technological advances. The development and standardization of molecular biology methods have led to faster detection and identification of a) Mycobacteria in clinical samples and b) resistance genes for early diagnosis of MDR-TB for a rapid treatment [18].

An in-house real-time PCR was developed by Barletta F *et al*. [14] showing good sensitivity and specificity to detecting TB complex. This technique was only been tested on pulmonary samples. Being so, our study aimed to test the same technique on pulmonary and extra-pulmonary samples in hospital settings. For this purpose, we chose to work on samples with the cited inclusion criteria to increase the number of positive cases. We also evaluated the performance of this technique through trisodium phosphate and the modified Petroff's method. The choice of IS6110 as a target of our real-time PCR is due to its presence in all MTBC members with multiple copies in the genome [19], making it a good target for MTBC detection. The choice of trisodium phosphate as decontamination methods is related to its low contamination rates on LJ media, high recovery of MTB, and its intermediate cost compared to other decontamination techniques [20]. The agreement between real-time PCR and culture was (k = 0.840), which seems to be a strong value. Five samples turned out false negative, which is similar to the results described by Barletta F *et al*. [14]. This issue could be related to the unequal distribution of bacterial cells of sputum, pus samples and tissue biopsies [21, 22]. Some rare cases reported a lack of IS6110 sequence [23], and the PCR of IS6110 from culture (data not showed) shows the presence of the fragment in the strains.

On the other side, for culture-negative and RT- PCR-positive samples, 2/7 specimens showed a positive AFB smear and 2/4 positive culture of the same patients in other samples. 2/7 were on treatment for TB, and the last one 1/7 was a BCGite case confirmed clinically (Figure 1). Positive PCR responses can be explained by its high sensitivity detecting small amounts of DNA, unlike culture on LJ media which detects only 10-100 viable mycobacteria/ml [14, 24]. On the other hand, DNA can be detected from non-cultivable or dead MTC bacteria for the cases treated [25]. Therefore, our study confirms that 1) the use of culture or the PCR alone cannot be a criterion to determine the negativity of the results and 2) a comparison with the clinical data is strongly necessary when making decisions during diagnosis [26]. Real-time PCR showed a high sensitivity (95.2%) and a good specificity (95.4%) on pulmonary samples, notice that the same rates were reported by other authors [14, 27]. For extra-pulmonary samples, sensitivity was lower (72%), but it was close to those reported elsewhere [28]. The literature has shown a sensitivity rate ranging from 37% to 96%, depending on the smear result and specimen type [24]. This could be explained by the low bacillary load in extra-pulmonary samples, unequal distribution load, and the presence of PCR inhibitors [29].

The limitation of this study is related to the sample number. The present work covers 11 non-respiratory positive specimens only, which is not very ideal to measure the performance of this technique and draw generalizable conclusions. For this reason, future projects could aim to develop this technique on exclusively extra-pulmonary samples. This technique is a promising auxiliary tool for diagnosing active pulmonary and extra-pulmonary TB when used in conjunction with other tests, seeking to increase the sensitivity and specificity of the diagnosis. Besides, this study demonstrates the first use of real-time PCR Taqman in Morocco for MTB detection. This technique showed it effectiveness compared to standard techniques on pulmonary and extra pulmonary samples. We suggest that multicentric studies be carried out in the future to assess whether this technique is affordable, achievable and reliable for routine diagnoses.

## Conclusion

The implementation of molecular approaches for direct diagnosis of TB, as part of the routine analysis in laboratories of health care institutions, will be of great use when adapting treatment regimens and limiting the dissemination of TB strains. This is very likely to ensure better management of TB in Morocco.

### What is known about this topic

- Extrapulmonary TB is relatively high (around 50% of cases) in Morocco;
- Conventional techniques have variable sensitivity and are time-consuming;
- Molecular techniques show a good sensitivity on pulmonary samples.

### What this study adds

- IS-6110 real-time PCR can work with different decontamination techniques (trisodium phosphate and modified Petroff methods);
- Real-time PCR showed good sensitivity and specificity for MTB detection on extra-pulmonary specimens;
- Solid culture is not sufficient to make a diagnosis of TB and should be associated with clinical data.

## References

[1] WHO Tuberculosis country profiles.

[2] Royaume du Maroc - Ministère de la Santé. Bulletin Epidémiologique.

[3] Steingart KR, Ng V, Henry M, Hopewell PC, Ramsay A, Cunningham J (2006). Sputum processing methods to improve the sensitivity of smear microscopy for TB: a systematic review. Lancet Infect Dis.

[4] Tyrrell FC, Budnick GE, Elliott T, Gillim-Ross L, Hildred MV, Mahlmeister P (2012). Probability of Negative Mycobacterium TB Complex Cultures Based on Time to Detection of Positive Cultures: a Multicenter Evaluation of Commercial-Broth-Based Culture Systems. J Clin Microbiol.

[5] Hida Y, Hisada K, Shimada A, Yamashita M, Kimura H, Yoshida H (2012). Rapid Detection of the Mycobacterium TB Complex by Use of Quenching Probe PCR (geneCube). J Clin Microbiol.

[6] Lira LAS, Santos FCF, Carvalho MSZ, Montenegro RA, Lima JFC, Schindler HC (2013). Evaluation of a IS6110-Taqman real-time PCR assay to detect Mycobacterium TB in sputum samples of patients with pulmonary TB. J Appl Microbiol.

[7] Ling DI, Flores LL, Riley LW, Pai M (2008). Commercial Nucleic-Acid Amplification Tests for Diagnosis of Pulmonary TB in Respiratory Specimens: Meta-Analysis and Meta-Regression. PLoS One.

[8] Moure R, Munoz L, Torres M, Santin M, Martin R, Alcaide F (2011). Rapid Detection of Mycobacterium TB Complex and Rifampin Resistance in Smear-Negative Clinical Samples by Use of an Integrated Real-Time PCR Method. J Clin Microbiol.

[9] Walusimbi S, Bwanga F, De Costa A, Haile M, Joloba M, Hoffner S (2013). Meta-analysis to compare the accuracy of GeneXpert, MODS and the WHO 2007 algorithm for diagnosis of smear-negative pulmonary TB. BMC Infect Dis.

[10] CDC Tuberculosis (TB) Disease: Symptoms and Risk Factors.

[11] Vandepitte J, Engback K, Piot P, Heock C (1991). Basic laboratory bacteriology.

[12] O Diraa, K Fdany, M Boudouma, N Elmdaghri, M Benbachir (2003). Assessment of the Mycobacteria Growth Indicator Tube for the bacteriological diagnosis of TB. Int J Tuberc Lung Dis.

[13] Doig C, Seagar AL, Watt B, Forbes KJ (2002). The efficacy of the heat killing of Mycobacterium TB. J Clin Pathol.

[14] Barletta F, Vandelannoote K, Collantes J, Evans CA, Arévalo J, Rigouts L (2014). Standardization of a TaqMan-based real-time PCR for the detection of Mycobacterium TB-complex in human sputum. Am J Trop Med Hyg.

[15] (2007). DELM «Direction de l´épidémiologie et de lutte contre les maladies?» Epidemiologic bulletin. Kingdom of Morocco.

[16] Tazi L, El Baghdadi J, Lesjean S, Locht C, Supply P, Tibayrenc M (2004). Genetic Diversity and Population Structure of Mycobacterium TB in Casablanca, a Moroccan City with High Incidence of TB. J Clin Microbiol.

[17] Lange C, Mori T (2010). Advances in the diagnosis of Tuberculosis. Respirology.

[18] Khosravi AD, Goodarzi H, Alavi SM, Akhond MR (2014). Application of Deletion-Targeted Multiplex PCR technique for detection of Mycobacterium TB Beijing strains in samples from TB patients. Iran J Microbiol.

[19] Alonso H, Samper S, Martín C, Otal I (2013). Mapping IS6110 in high-copy number Mycobacterium TB strains shows specific insertion points in the Beijing genotype. BMC Genomics.

[20] Chatterjee M, Bhattacharya S, Karak K, Dastidar SG (2013). Effects of different methods of decontamination for successful cultivation of Mycobacterium TB. Indian J Med Res.

[21] Burkardt H-J (2000). Standardization and Quality Control of PCR Analyses. Clin Chem Lab Med.

[22] Kesarwani RC, Pandey A, Misra A, Singh AK (2004). Polymerase chain reaction (PCR): Its comparison with conventional techniques for diagnosis of extra-pulmonary tubercular diseases. Indian Journal of Surgery.

[23] el Baghdadi J, Lazraq R, Benani A, Naciri M, Ibrahimy S, Benslimane A (1997). [PCR detection of Mycobacterium TB lacking IS 6110]. Bull Soc Pathol Exot.

[24] Babafemi EO, Cherian BP, Banting L, Mills GA, Ngianga K (2017). Effectiveness of real-time polymerase chain reaction assay for the detection of Mycobacterium TB in pathological samples: a systematic review and meta-analysis. Syst Rev.

[25] Bloemberg GV, Voit A, Ritter C, Deggim V, Böttger EC (2013). Evaluation of Cobas TaqMan MTB for Direct Detection of the Mycobacterium TB Complex in Comparison with Cobas Amplicor MTB. J Clin Microbiol.

[26] Siala M, Smaoui S, Taktak W, Hachicha S, Ghorbel A, Marouane C (2017). First-time detection and identification of the Mycobacterium TB Complex members in extrapulmonary TB clinical samples in south Tunisia by a single tube tetraplex real-time PCR assay. PLoS Negl Trop Dis.

[27] Sadr S, Darban-Sarokhalil D, Irajian GR, Imani Fooladi AA, Moradi J, Feizabadi MM (2017). An evaluation study on phenotypical methods and real-time PCR for detection of Mycobacterium TB in sputa of two health centers in Iran. Iran J Microbiol.

[28] Causse M, Ruiz P, Gutiérrez-Aroca JB, Casal M (2011). Comparison of Two Molecular Methods for Rapid Diagnosis of Extrapulmonary TB. J Clin Microbiol.

[29] Armand S, Vanhuls P, Delcroix G, Courcol R, Lemaître N (2011). Comparison of the Xpert MTB/RIF Test with an IS6110-TaqMan Real-Time PCR Assay for Direct Detection of Mycobacterium TB in Respiratory and Nonrespiratory Specimens. J Clin Microbiol.

